# Dissipation of Energy by Dry Granular Matter in a Rotating Cylinder

**DOI:** 10.1038/srep26833

**Published:** 2016-06-03

**Authors:** Achim Sack, Thorsten Pöschel

**Affiliations:** 1 Institute for Multiscale Simulation, Nägelsbachstraße 49b, 91052 Erlangen, Germany

## Abstract

We study experimentally the dissipation of energy in a rotating cylinder which is partially filled by granular material. We consider the range of angular velocity corresponding to continous and stationary flow of the granulate. In this regime, the stationary state depends on the angular velocity and on the filling mass. For a wide interval of filling levels we find a universal behavior of the driving torque required to sustain the stationary state as a function of the angular velocity. The result may be of relevance to industrial applications, e.g. to understand the power consumption of ball mills or rotary kilns and also for damping applications where mechanical energy has to be dissipated in a controlled way.

The flow of granular material inside a rotating cylinder is of great technological importance and has been subject to research for many years. In the effort to fully understand the static and dynamical behaviour of such a system, a surprising variety of physical phenomena has been discovered: Depending on angular velocity, diameter of the cylinder, filling level, friction between particles and the wall, and particle material characteristics, different regimes of granular flow are observed which have been termed^1^ slipping, slumping, rolling, cascading, cataracting, and centrifuging. Except for *slipping*’ where the granulate assumes a solid state and slides against the wall, these regimes can be observed in dependence on rotation velocity while all other parameters are fixed.

For low angular velocity, the flow is non-stationary (slumping) and one observes avalanches of characteristic size distribution^2^,^3^,^4^,^5^,^6^. Correspondingly, the free surface of the granulate forms a plane whose tilt fluctuates between the angle of marginal stability and the angle of repose^7^,^8^,^9^,^10^,^11^,^12^. When increasing the angular velocity, the time lag between successive avalanches shrinks until it approaches the duration of avalanches such that the discrete avalanche-like flow turns into continuous flow (rolling). In rolling mode, Fig. 1a the granulate reveals a flat free surface tilted by a characteristic angle called *dynamic angle of repose* being a function of the angular velocity, e.g.^13^,^14^,^15^,^16^. The transition between the slumping and rolling regimes is under scientific debate since almost a century^17^,^18^: While some references^14^,^19^,^20^ report a hysteretic transition or co-existence^21^ of both regimes in a certain interval of rotation velocity, others do not see hysteresis^22^ but intermittent behavior^23^. When further increasing the rotation velocity, the systems passes into the cascading regime, Fig. 1b, where the system develops a characteristic *S*-shaped surface profile^9^,^24^,^25^. Both rolling and cascading regimes develop a characteristic bulk flow profile which in turn gives rise to important effects like convection, mixing, segregation and others. Therefore, the flow profile was subject of intensive experimental and theoretical research, e.g.^20^,^26^,^27^,^28^,^29^,^30^,^31^,^32^,^33^,^34^ and many others. Particularly detailed experimental results can be found in refs 35,36. For yet larger angular velocity, we arrive at the regime of cataracting, Fig. 1c where part of the flow gets airborne and moves along ballistic trajectories. From Fig. 1c we recognize an interesting structure of the flow, namely, the interface where the free falling particles hit the bulk material is still approximately plane revealing a certain inclination similar to the dynamic angle of repose. Finally, at very large angular velocity, centrifugal forces become dominant and the relative motion between the particles ceases, Fig. 1d. We wish to mention that under certain conditions, for very slow angular velocity such that only a very thin layer flows, of width of the order of a particle diameter, there exists a further dynamical regime leading to *polydirectional stable bulk material*^37^. In this regime, the flow is interrupted by rare stochastic collapse events, thus, in strict sense this regime does not qualify as a stationary state. For details see^37^.

While monodisperse granular material in rotating cylinders shows complicated dynamics, it becomes even more complex when the material reveals noticeable polydispersity which gives rise to various effects of mixing/segregation and self-organized structure formation. Remarkably, structure formation occurs in radial *and* azimuthal direction. For a detailed discussion see^12^,^18^,^38^.

In the present paper, we consider the motion of granular material in a rotating cylinder in the range of angular velocity large enough to provide continous flow and small enough such that the system is not dominated by centrifugal force. In this regime, the granular flow is stationary, that is, independent of time and its properties depend on the angular velocity and on the volume fraction. In particular, we are interested in the dissipation of energy due to the inelastic nature particle-particle and particle-wall interaction of the particles. Consequently, we aim to an understanding of the dissipative properties of such systems. Obviously, when granular material is agitated by an external force, e.g. by a linear vibration, energy is dissipated which is the basic idea of granular dampers which have been investigated recently^39^,^40^,^41^,^42^,^43^,^44^,^45^,^46^. Similarly, when a cylinder partially filled by granular material rolls down an inclined plane, the rolling motion is damped due to the motion of the granulate^47^,^48^,^49^, which may be considered as a damper for rotational motion. In the following Sections we will elaborate the mechanics of energy dissipation in rotating cylinders filled by granular matter in detail.

## Results

### Dissipation as a Function of Angular Velocity

**Figure 1:**
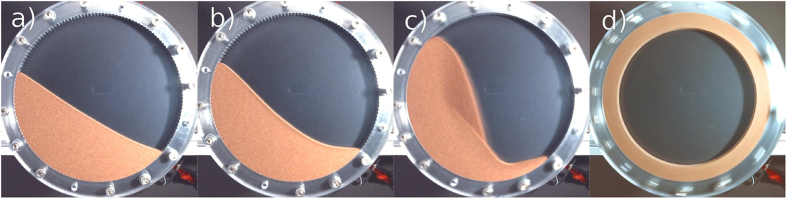
Regimes of stationary continuous granular flow for different rotational velocity: (**a**) rolling (0.314 rad/s), the free surface is flat and tilted by a characteristic angle; (**b**) cascading (0.942 rad/s), the typical S-shape develops; (**c**) cataracting (6.91 rad/s), part of the flow gets airborne and moves along ballistic trajectories. Because of lower density, the free-falling granulate appears slightly darker. The interface where the free falling particles hit the bulk material is approximately plane in this regime; (**d**) centrifuging (21.36 rad/s), the relative motion of the particles ceases.

**Figure 2:**
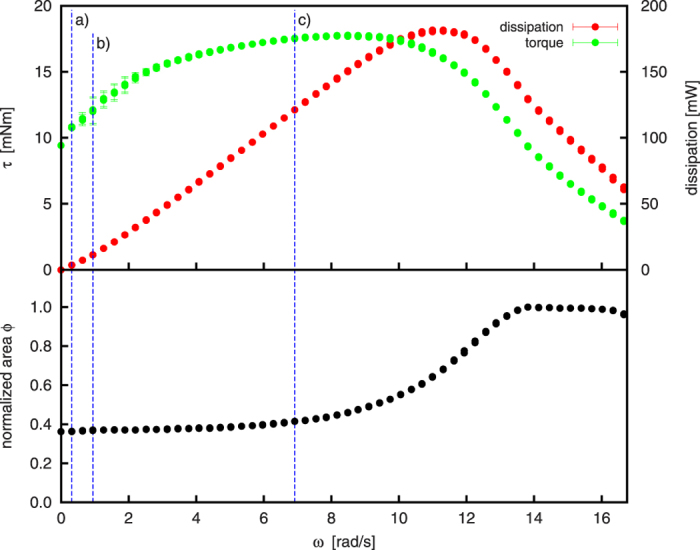
Torque, *τ*, dissipated power, and normalized area of the cylinder cross section populated by granulate, as seen by the camera, for the system shown in Fig. 1 (sample No. 6). Where not explicitly shown, the errorbars are smaller than the symbol size. Vertical dashed lines indicate the angular velocities corresponding to Fig. 1a–c.

The introducing example system, Fig. 1, shows a rotating cylinder of diameter 100 mm and length 5 mm filled by 70 g bronze powder corresponding to a volume fraction of *φ* = *V*_granulate_/*V*_cylinder_ = 0.34. This sample is referred to as “sample No. 6” in the subsequent text. The details of the experimental setup, the specification of the granulate and the protocol of measurement can be found in Sec. “Methods”. For this system, Fig. 2 shows the full data available from our experiment, namely

- The torque exerted by the motor to the cylinder to compensate for the loss of energy due to dissipative dynamics of the granulate inside the cylinder,
- The corresponding power dissipated by the granulate,
- The area of the cylinder cross section populated by granulate, as seen by the camera. The value is normalized by the visible area of the whole cylinder, i.e., *ϕ* = 1 for a cylinder completely filled by granulate.

The vertical dashed lines in Fig. 2 indicate the angular velocities corresponding to Fig. 1a–c showing the characteristic regimes of stationary granular flow.

For angular velocity 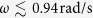, the material is found in rolling regime where the free boundary of the granulate is nearly flat, see Fig. 1a). For 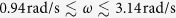, the system is in cascading regime where the free surface assumes its characteristic S-shape (Fig. 1b). In this regime, the flow of granulate is bound to the curved surface. Consequently, for *ω* < 3.14 rad/s, the visible area is almost constant up to a small inflation due to Reynolds dilatancy. With increasing *ω*, the granulate moves to the left and upwards. This shift of the center of mass causes an increase of the torque which is necessary to turn the cylinder at the given velocity.

For further increased angular velocity, 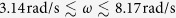, successively more and more granulate gets dragged along the upward-moving wall due to centrifugal forces. This leads to a further shift of the center of mass to the left and the torque exerted by the granulate increases. If the rotational speed is larger than approximately 4.1 rad/s, the particles of largest velocity, i.e. those located in the middle of the continuous surface flow, perform ballistic trajectories which progressively results in a broader stream of airborne particles, that is, the system is in the cataracting regime. Due to an increasing portion of particles performing ballistic trajectories, the bulk of granular material in the S-like shape is reduced with increasing *ω*. As a consequence, the torque reaches its maximum at *ω* ≈ 8.48 rad/s and decreases for larger *ω*. For 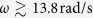, centrifuging occurs, and beginning with the outermost layer, more and more granulate is held to the cylinder wall by the centrifugal force. For *ω* > 21.4 rad/s all granulate centrifuges. At this point, the granulate is distributed evenly on the perimeter of the drum with the center of gravity exactly on the rotation axis and the torque drops to zero. This agrees with the fact that in the centrifuging regime the particles do not move with respect to one another and, thus, no energy is dissipated which must be balanced by the motor.

From the bottom graph of Fig. 2 showing the fraction *ϕ* of the area covered by the granulate as seen by the camera, we find that 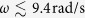, the granulate is rather compact since the occupied area is nearly constant. With increasing velocity, more and more particles get airborne, the overall density of the granulate decreases and the occupied area increases, until *ω* ≈ 13.6 rad/s when the whole cylinder is filled by cataracting granulate, i.e. *ϕ* = 1. For yet larger velocity, 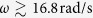 (not shown in Fig. 2), *ϕ* decreases since an increasing part of the material is held to the outer wall of the cylinder by centrifugal forces. For 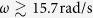, we observe unsteady and irregular behavior of all three curves shown in Fig. 2. The reason for this more complex dynamics is not clear yet and shall not be discussed in this paper.

### Dissipation as a Function of  Volume Fraction

**Figure 3:**
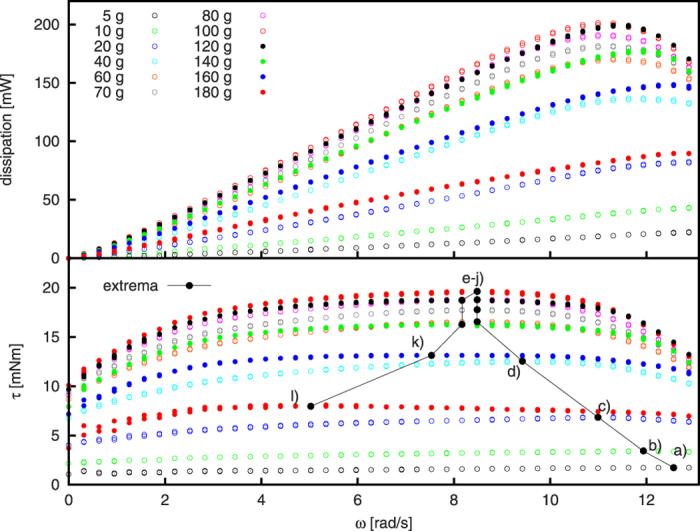
Dissipated power and torque for the samples specified in Table 1 as a function of the angular velocity. At fixed frequency, the torque and, thus, the dissipation increase with increasing filling mass for 5 g < *m* ≤ 100 g. For *m* > 100 g (more than half filled cylinder), the torque and dissipation decrease with increasing mass. The torque reveals a plateau of approximately constant torque for 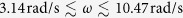. For each curve, the frequency corresponding to maximal torque is marked by black circles connected by lines to guide to the eye. The labels a) - l) relate to the snapshots shown in Fig. 6.

To investigate the effect of filling level on the dissipation, we repeat the experiments with different amount of granular material, *m* = (5 … 180) g corresponding to volume fraction *φ* = (0.024 … 0.877). The set of performed experiments is specified in Table 1. For each setup, the same procedure as for sample No. 6 was followed, see Sec. “Methods” for details. The torque exerted by the motor to the cylinder and the dissipation of the granulate for each setup is shown in Fig. 3.

Figure 3 shows, that for an increasing mass up to *m* = 100 g (half filled cylinder), the torque and thus the dissipation increases for a fixed velocity. This is explained by the larger mass of flowing granular material which undergoes more particle-particle collisions and thus dissipates more energy. However, for more than a half filled cylinder, i.e. *m* > 100 g, the progressively constrained space hinders the granulate to flow freely, thus torque and dissipation declines. For 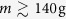, a dead region of material forms around the axis of the cylinder (see, e.g.^6^). The material in this region rotates in synchrony with the cylinder, however, the particles in this region do not move with respect to each other, thus, these particles do not contribute to dissipative flow. Consequently, with increasing volume fraction, the torque decreases.

For all volume fractions shown in Fig. 3, the torque reveals a plateau-like region at 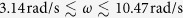. In the next section we describe a model which allows to scale the data such that the curves collaps for a filling *m* = (40 … 160) g, that is, for data sets 4–11 described in Table 1.

### Limit of Slow Rotation

**Figure 4:**
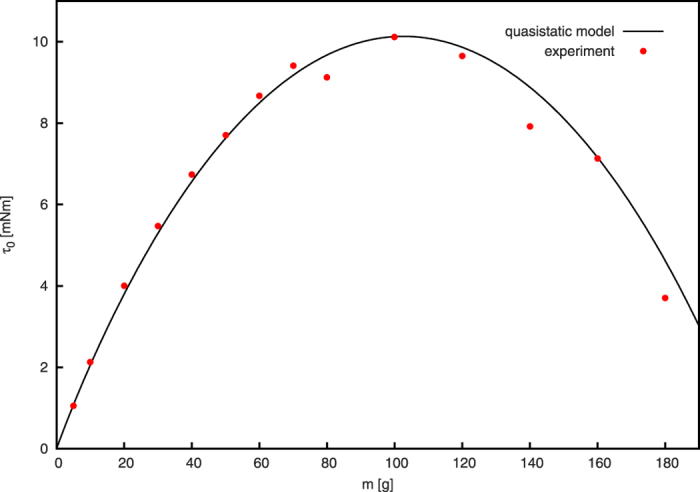
Torque as a function of the filling mass *m* in the limit of small angular velocity, *ω* → 0.

In the limit of slow rotation the inclination of the granulate is determined by the static angle of repose. In this limit, we can compute the torque as a function of the mass. See Sec. “Methods” for the details of the model. Figure 4 shows the torque as a function of the filling mass as obtained from the experiment in comparison with the model in very good agreement. For small volume fraction, the torque increases approximately linearly. It reaches its maximum when the container is half filled, *φ* = 0.5, and decreases for yet higher volume fraction. Obviously, for *φ* = 1, the torque vanishes.

The experimental data shown in Fig. 4 was obtained after each frequency sweep by gently stopping the driving motor and recording a final measurement. The good agreement of the experimental data and the model, justifies the assumptions of the static model, see Sec. “Methods” for discussion.

### Normalization of the experimental data

**Figure 5:**
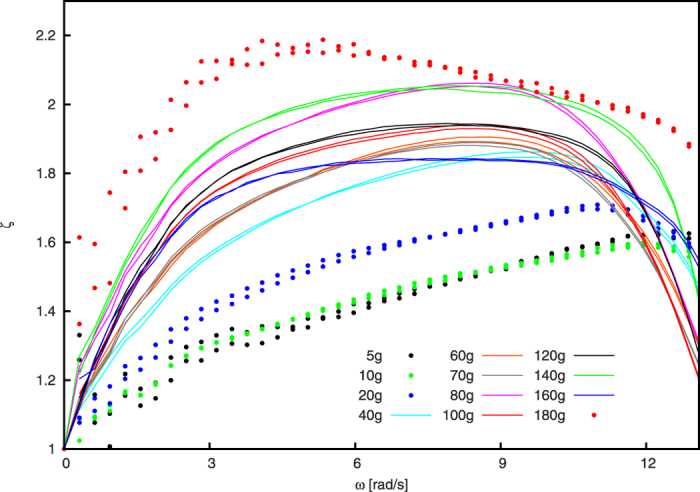
Torque as a function of angular velocity. The figure shows the same data as Fig. 3 but normalized by the corresponding quasistatic limit, *ζ* ≡ *τ*/*τ*_0_.

When we normalize the data shown in Fig. 3 by the corresponding quasistatic limit, *ζ* ≡ *τ*/*τ*_0_, see Fig. 5, the curves in a wide range of lilling ratio, *m* = (40 … 160) g, show very similar behaviour (lines in Fig. 5). For these samples, the maximum torque is assumed for the same angular velocity, *ω* ≈ 8.48 rad/s. For *m* < 40 g, the maximal torque is reached for larger angular velocity, whereas for fillings *m* > 160 g, the torque peaks at lower values, see also Fig. 3.

**Figure 6:**
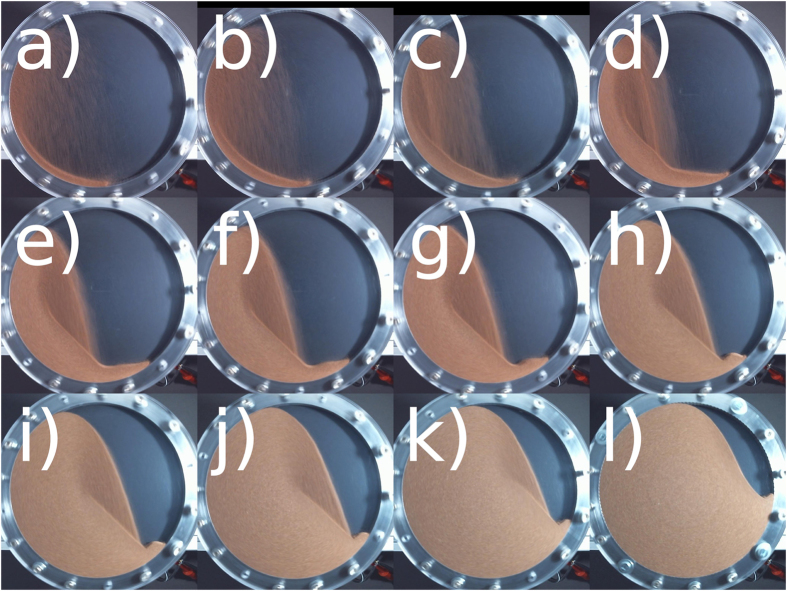
Snapshots of the cylinder at different filling levels, rotating at the frequency corresponding to the maximal torque. Images (**a**–**l**) correspond to the points labelled in Fig. 3. For *m* = (40 … 160) g (**d**–**k**), the bulk of material where the jet of airborne particles impact, exposes a flat plane of approximetely identical slope.

While we do not have an explanation for the collaps of the data for *m* = 40 … 160 g, we find the dynamics of the granulate phenomenologically similar in all those cases, see Fig. 6: The interface where the stream of airborne particles hits the bulk of material is nearly a plane with constant slope, *α* ≈ 42° with respect ot the horizontal plane, see Fig. 6d–k. This angle is notably different from the angle of repose. For lower fillings (a–c), there is not enough material available for the bulk to form this line. For higher volume fraction (k), a co-rotating center develops where the material does not take part in the cataracting motion. For a nearly full disk the granulate does not get airborne at all, Fig. 6(l).

## Conclusion

The torque exerted by granular material flowing inside a rotating cylinder was measured for a wide range of volume fraction. For small angular velocity, we propose a quasistatic model whose results are in good agreement with the experiment. When the experimentally obtained torque is scaled by the quasistatic limit, *ζ* = *τ*/*τ*_0_, we obtain a collaps of the measured data for a wide range of volume fraction. In this interval, the granulate reveals similar phenomenological behavior. In earlier numerical studies by Dragomir *et al.*^49^ it was found that the dissipation of energy increases linearly with the rotation velocity. This is in contradiction with our experimental results showing a characteristic dependence of the torque due to dissipation on the angular velocity which disagrees with a linear increase of energy dissipation.

## Methods

### Experimental Setup

**Figure 7:**
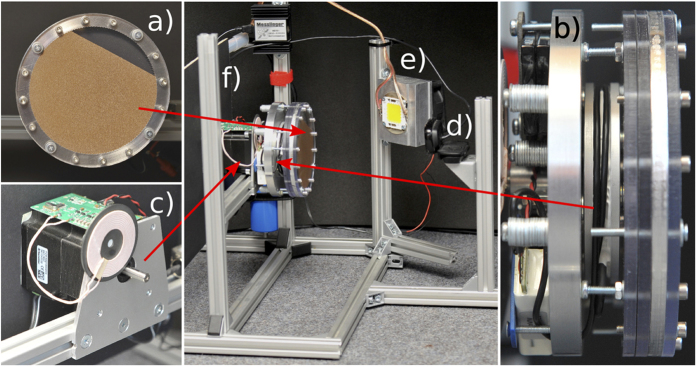
Experimental setup: (**a**) short cylinder partially filled by granular material, (**b**) torque-measurement unit, (**c**) driving servo motor, (**d**) camera, (**e**) illumination unit, (**f**) aluminum rack, and (**g**) a computer for the control and data recording (not shown).

The experimental setup shown in Fig. 7 consists of a aluminum rack carrying the servo motor, the illumination unit and the camera. The motor drives an assembly consisting of the torque-measurement unit and the cylindrical container partly filled by granular material. A computer is used for the control of the servo motor and for data recording.

**Figure 8:**
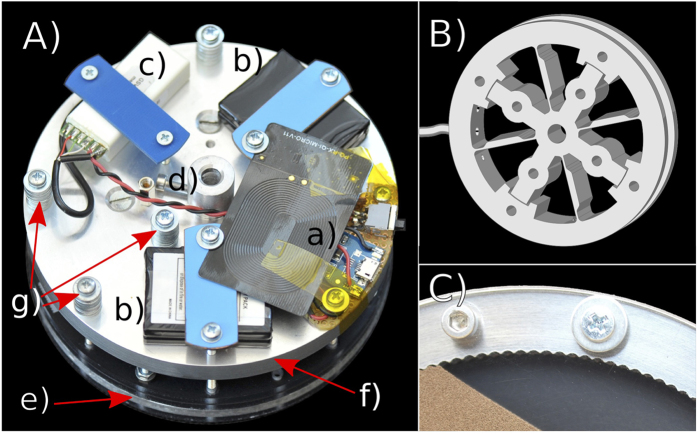
Details of the experimental setup. (**A**) torque-measuring unit, consisting of (**e**) the cylinder partly filled by granulate and (**f**) the aluminium mount with the motor coupling (**d**). The mount carries the radio link (**c**), balance weights (**g**), the batteries (**b**) and charging circuitry (**a**). The torque sensor, sketch (**B**), is located between the mount (**f**) and the cylinder (**e**). (**C**) detail of Fig. 7a showing the grooves at the inner side of the cylinder.

The cylindrical container (Fig. 7a) is built from a ring with inner radius 50 mm and thickness 5 mm forming the curved surface of the cylinder. The ring is milled from aluminium and in order to avoid slippage of the granulate, its inner side is roughened by means of half-cylindrical grooves of 1 mm radius, see Fig. 8C. The flat sides of the cylinder are made from polycarbonate plates of 4 mm thickness whose inner sides are ITO-coated to avoid electrostatic charges. Front plane, back plane and aluminum ring are held together by screws to form the cylindrical container. The cylinder is coupled to a motor flange via the torque-measuring unit, Fig. 8A. This unit contains a torque sensor, Fig. 8B, consisting of an inner hub which is connected by thin, flexible spokes with strain gauges to an outer mounting ring. When a torque is applied between the mounting ring and the hub, the spokes deform slightly and change the signal from the strain gauges. To dampen the torsional vibration at about 3.14 rad/s resulting from the low stiffness of the sensor and the cogging torque of the servo motor, a small amount of Nye Flourcarbon 868 H damping grease was added in a slit between the opposing faces of the sensor, such that it bypasses the force sensor and dampens vibrations. A comparison of the damped and the non-damped system showed that the damping had no influence on the sensitivity and precision of the sensor. The outer ring of the torque sensor is fixed to a mount which carries the motor flange, which is then mounted on the shaft of a computer controlled servo motor, see Fig. 7c. Further, the torque-measuring unit is equipped with a battery powered radio link to transmit the torque sensor data to a recording PC for later processing. The radio link is necessary, since the torque sensor itself rotates with the granulate filled disk driven by the servo motor. To charge the battery during standstill, an inductive power transmitter transmitts energy to the torque-measuring unit. In empty state, the torque measuring unit was carefully balanced by means of balance weights attached to the mount. A camera provides still images of the flow pattern inside the disk.

### Specification of the Granulate

For the experiment we used monodisperse phosphor bronze powder (type 83PP Bronze from ACuPowder International, LLC) with particle diameter 150 *μ*m (bulk density 5,230 kg/m^3^, angle of repose 28.3°). The size of the particles was chosen to be small compared to the length of the cylinder, but large enough for humidity and electrostatics to be neglegible. In combination with the conductive coating of the plane walls of the cylinder and the spacer ring made from aluminum, we did not observe any sign of electrostatic effects.

### Protocol of the Measurement and Data Processing

In preparation of measurements, the cylinder was carefully cleaned and then filled by the granlate with a volume fraction of


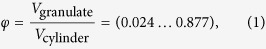


where *V*_granulate_ and *V*_cylinder_ is the volume occupied by the granulate and the volume of the empty cylinder respectively. During the run of a measurement, the rotational velocity of the motor was first increased, *ω* = 0.31 rad/s…*ω*_max_ in steps of Δ*ω* = 0.31 rad/s and then decreased likewise to standstill. For each volume fraction, *ω*_max_ was chosen such that for all velocities a steady state was assumed. For rotational velocities larger than *ω*_max_, unsteady and irregular behavior was observed, possibly with long-lived transients. The more complex behavior appearing for *ω* > *ω*_max_ will be described elsewhere and shall not be subject of the present paper.

For frequencies, *ω* ≤ *ω*_max_, we did not observe long transients nor hysteretic behavior, therefore, when changing the rotational velocity it was sufficient to wait for 2 seconds to allow transients to die out, before recording the data from the torque sensor. For each value of *ω* we recorded torque data for 10 seconds at a sample rate of 100 sps. From the acquired data, the mean, *τ*, and standard deviation of the torque were calculated yielding finally the average power *P* that is dissipated by the granulate inside the rotating cylinder,


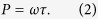


For each frequency step, 10 pictures of the experiment were taken by a camera, in order to

- Obtain a visual impression of the flow pattern inside the rotating cylinder,
- Identify unsteady flow by numerically comparing pictures belonging to the same value of *f*,
- Compute the region populated by granulate inside the cylinder.

Using a circular mask the image was first cropped along the inner race of the aluminum ring such that only the inside of the disk was left on the picture. The number of pixels *N*_*D*_ remaining in the picture correspond to the total area of the disk. Then, using an algorithm programmed in Matlab, the number of pixels in the cropped picture with the same colour as the bronze powder *N*_*B*_ were identified. The fractional area which is covered by the granulate is then obtained by:


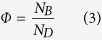


### Model for Quasistatic Rotation, *ω* → 0

Despite the full dynamics of the rotating cylinder being complicated even in the quasistatic case, for the limit of small angular velocity one can derive a rather realistic model, see Fig. 4. The limit *ω* → 0 is problematic since for very slow rotation, the granular flow is non-stationary^2^,^3^,^4^,^5^,^6^,^37^. Therefore, we have to be more specific: What we wish to describe is the torque exerted by *stationary flow* for slow rotation, thus, the angular velocity shall be small but large enough to sustain stationary flow.

**Figure 9:**
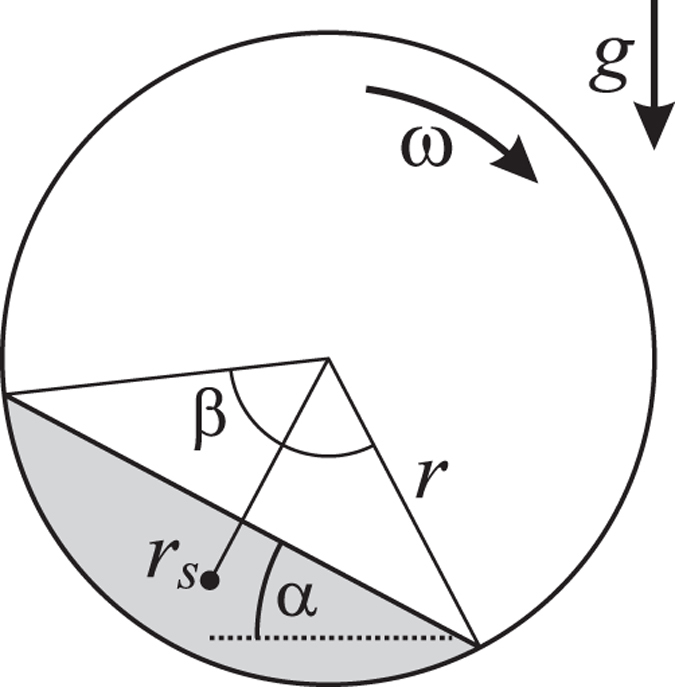
Sketch of the model: granular material (shaded area) resides in a segment of the drum characterized by *β*. *r*_*s*_ is the distance of the center of mass of the granulate from the center of rotation. *α* is the static angle of repose.

The model is sketched in Fig. 9: Granulate of mass *m* and density *ρ* occupies a segment of the cross-sectional circle of radius *r* (shaded area in Fig. 9). *α* is the angle of repose, and *d* is the width of the cylinder. The area of the segment is characterized by the angle *β*, see Fig. 9, which relates the mass by


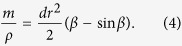


From geometry we obtain the distance of the center of mass of the granulate from the rotation axis,


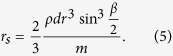


The torque follows from the assymmetry of the mass distribution due to the angle of repose, *α*,


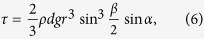


where *β* is the solution of Eq. (4). This model which does not contain any free parameters was used to compute the torque for small angular velocity. For comparison with experimental data see Fig. 4.

## Additional Information

**How to cite this article**: Sack, A. and Pöschel, T. Dissipation of Energy by Dry Granular Matter in a Rotating Cylinder. *Sci. Rep.* **6**, 26833; doi: 10.1038/srep26833 (2016).

## Figures and Tables

**Table 1:** Mass of granulate, *m*, and corresponding volume fraction, *φ*, for the performed experiments.

Sample No.	Mass *m* [g]	Volume fraction *φ*
1	5.00	0.024
2	9.97	0.049
3	20.00	0.097
4	39.99	0.195
5	60.00	0.292
6	70.01	0.341
7	80.01	0.390
8	100.00	0.487
9	120.01	0.585
10	140.00	0.682
11	160.00	0.779
12	180.00	0.877

Sample No. 6 corresponds to the case discussed in the previous sections, see Figs 1 and 2.
